# Dorsomedial prefrontal cortex activation disrupts Pavlovian incentive motivation

**DOI:** 10.3389/fnbeh.2022.999320

**Published:** 2022-10-13

**Authors:** Briac Halbout, Collin Hutson, Kate M. Wassum, Sean B. Ostlund

**Affiliations:** ^1^Department of Anesthesiology and Perioperative Care, School of Medicine, University of California, Irvine, Irvine, CA, United States; ^2^Department of Psychology, University of California, Los Angeles, Los Angeles, CA, United States; ^3^Department of Neurobiology and Behavior, School of Biological Sciences, University of California, Irvine, Irvine, CA, United States; ^4^UC Irvine Center for Addiction Neuroscience, School of Biological Sciences, University of California, Irvine, Irvine, CA, United States; ^5^Center for the Neurobiology of Learning and Memory, School of Biological Sciences, University of California, Irvine, Irvine, CA, United States

**Keywords:** motivation, DREADD, anterior cingulate, behavioral flexibility, cognitive control

## Abstract

The dorsomedial prefrontal cortex (dmPFC) is known to make important contributions to flexible, reward-motivated behavior. However, it remains unclear if the dmPFC is involved in regulating the expression of Pavlovian incentive motivation, the process through which reward-paired cues promote instrumental reward-seeking behavior, which is modeled in rats using the Pavlovian-instrumental transfer (PIT) task. The current study examined this question using a bidirectional chemogenetic strategy in which inhibitory (hM4Di) or excitatory (hM3Dq) designer G-protein coupled receptors were virally expressed in dmPFC neurons, allowing us to later stimulate or inhibit this region by administering CNO prior to PIT testing. We found that dmPFC inhibition did not alter the tendency for a reward-paired cue to instigate instrumental reward-seeking behavior, whereas dmPFC stimulation disrupted the expression of this motivational influence. Neither treatment altered cue-elicited anticipatory activity at the reward-delivery port, indicating that dmPFC stimulation did not lead to more widespread motor suppression. A reporter-only control experiment indicated that our CNO treatment did not have non-specific behavioral effects. Thus, the dmPFC does not mediate the expression of Pavlovian incentive motivation but instead has the capacity to exert pronounced inhibitory control over this process, suggesting that it is involved in adaptively regulating cue-motivated behavior.

## Introduction

Pavlovian reward-associated cues acquire potent motivational properties which allow them to instigate instrumental reward-seeking behavior, a phenomenon selectively modeled by the Pavlovian-instrumental transfer (PIT) task (Cartoni et al., 2016; Corbit and Balleine, 2016). This motivational influence is normally adaptive, promoting the pursuit of goals like palatable food in situations where they are likely to become available. However, in substance use disorder and related conditions, cues can trigger intense cravings that motivate reward seeking even when efforts are made to abstain from such behavior (Unnithan et al., 1992; O’Brien et al., 1998; Sinha and Li, 2007; Tiffany and Wray, 2012; Fatseas et al., 2015; Vafaie and Kober, 2022). This maladaptive influence of cues is thought to be mediated, at least in part, by a loss of top-down inhibitory control over motivated behavior (Kober et al., 2010; Belin et al., 2013; Fatseas et al., 2015; Marshall and Ostlund, 2018; Antons et al., 2020).

The neural circuitry responsible for regulating cue-motivated behavior is not well understood, though the dorsomedial prefrontal cortex (dmPFC) is likely to be involved. The dmPFC, which refers here to the dorsal part of the prelimbic cortex as well as the anterior cingulate cortex, is richly connected with several brain regions known to mediate PIT (Cartoni et al., 2016; Corbit and Balleine, 2016), such as the nucleus accumbens, mediodorsal thalamus, and amygdala (Gabbott et al., 2005; Hoover and Vertes, 2007). Neural activity in the dmPFC is also strongly modulated by reward-predictive cues (Shidara and Richmond, 2002; Kennerley et al., 2011; Monosov, 2017), including during PIT testing (Homayoun and Moghaddam, 2009). Nevertheless, previous studies have found that disrupting dmPFC function does not alter PIT performance (Cardinal et al., 2003; Corbit and Balleine, 2003; Halbout et al., 2019), suggesting it may not be critical for the expression of Pavlovian incentive motivation.

However, the dmPFC has been strongly implicated in multiple aspects of behavioral flexibility including set-shifting (Ragozzino et al., 1999; Stefani et al., 2003; Floresco et al., 2006, 2008; Bissonette and Roesch, 2015; Powell and Redish, 2016; Brockett et al., 2020) and response inhibition (Bussey et al., 1996; Muir et al., 1996; Narayanan and Laubach, 2006; Jonkman et al., 2009; Terra et al., 2020; Hamel et al., 2022). Such studies have revealed that the dmPFC is important for withholding or otherwise modifying learned motor behaviors (e.g., instrumental habits), but do not directly address its role in negatively regulating Pavlovian incentive motivation as measured by PIT.

We hypothesized that the dmPFC is not required for the expression of PIT but does have the capacity to negatively regulate—or suppress—this motivational effect. To test this, we applied a bidirectional chemogenetic strategy. We reasoned that if the dmPFC is selectively involved in regulating Pavlovian incentive motivation, then activating this structure should attenuate PIT expression (i.e., it should dampen cue-motivated reward seeking). Furthermore, if one assumes that the PIT effect represents an adaptive motivational response to reward-paired cues, and is therefore normally expressed in an unregulated manner, then inhibiting the dmPFC should have little or no effect on this response. In contrast, if the dmPFC is more directly involved in mediating the expression of cue-motivated reward seeking, then inhibiting this structure should disrupt the PIT effect.

## Materials and methods

### Animals

Male Long-Evans rats (*N* = 26) were obtained from Charles River and weighed > 290 g at the start of the study. Female rats were not used here to minimize variability, as previous studies have observed significant sex differences in reward consumption (Marshall et al., 2017; Westbrook et al., 2018) and assays of incentive motivation (Pitchers et al., 2015; Reichelt et al., 2016; Madayag et al., 2017; Tapia et al., 2019) including PIT (Shields and Gremel, 2021; Derman and Lattal, 2022). Rats were paired-housed in transparent plastic cages in a temperature- and humidity-controlled vivarium. The rats were tested during the light phase of a standard 12:12 h light:dark schedule. Rats had *ad libitum* access to water in their home cages, and were food restricted (∼13.5 g/day of home chow; Envigo) to maintain them at between 85 and 90% their free-feeding bodyweight throughout the experiment. All experimental procedures were approved by the UC Irvine Institutional Animal Care and Use Committee (IACUC) and conducted in accordance with the National Research Council Guide for the Care and Use of Laboratory Animals.

### Apparatus

Operant behavioral procedures were conducted in identical operant chambers (ENV-007, Med Associates, St Albans, VT, USA), each housed in a sound- and light-attenuated cubicle. A food-delivery port was located at the center of one end-wall of the chamber, 2.5 cm above the stainless-steel grid floor. A cup within the food port was used to receive 45-mg grain pellets (BioServ) via an automated pellet dispenser. A photobeam detector positioned across the food-port entrance was used to monitor head entries. A retractable lever was positioned to the right of the food port. A houselight (3 W, 24 V) at the top of the opposite end-wall provided general illumination and a fan mounted on the cubicle provided ventilation and background noise. Experimental events were controlled and recorded with a 10-ms resolution using MED-PC IV software.

### Surgery

Rats were anesthetized using isoflurane and placed in a stereotaxic frame for microinjections of an adeno-associated virus (AAV) vectors to induce expression of the inhibitory DREADD [designer receptor exclusively activated by designer drug (Armbruster et al., 2007)] hM4Di [pAAV5-CaMKIIa-hM4D(Gi)-mCherry, 1.1 × 10^13^ vg/mL; Addgene plasmid # 50477-AAV5] (*n* = 8) or the excitatory DREADD hM3Dq [CaMKIIa-hM3D(Gq)-mCherry 1.7 × 10^13^ vg/mL; Addgene plasmid # 50476-AAV5] (*n* = 8) fused with mCherry under the CaMKII promoter. DREADDs are genetically modified G protein coupled receptors that can be selectively activated by the designer drug Clozapine-n-oxide (CNO) (Armbruster et al., 2007). While the activation of hM4Di results in a general silencing of neurons through neuronal hyperpolarization and presynaptic inhibition of neurotransmitters release (Armbruster et al., 2007), the activation of hM3Dq leads to enhanced firing of neurons by facilitating their depolarization (Alexander et al., 2009). The use of the CaMKII promoter allows for DREADD expression in putative excitatory cortical neurons (Dittgen et al., 2004; Nathanson et al., 2009). An AAV expressing only the fluorescent reporter protein GFP (AAV5-CaMKIIa-EGFP, 3.6 × 10^12^ vg/mL; Addgene plasmid # 50469-AAV5) was used in the control group (*n* = 10). The AAV was injected bilaterally into the dmPFC (+3.2 mm AP, ± 0.7 mm ML, –2.8 mm DV from bregma; 0.7 μL/side). Animals were allowed at least 5 days of recovery before undergoing food restriction and behavioral training. Testing occurred at least 25 days after surgery to allow adequate time for viral expression of hM4Di, hM3Dq, or GFP throughout dmPFC neurons.

### Pavlovian-instrumental transfer

#### Pavlovian conditioning

Rats first received 2 d of magazine training, during which 40 pellets were delivered into the food cup on a random 90-s intertrial interval (ITI). Rats then received 8 daily Pavlovian conditioning sessions. Each session consisted of a series of 6 presentations of a 2-min audio cue (CS+; either a pulsating 2 kHz pure tone (2 s at 80 db and 1 s at 90 db) or white noise; 80 dB), with trials separated by a 3 min variable ITI (range 2–4 min between CS onsets). During each CS+ trial, pellets were delivered on a 30-s random time schedule, resulting in an average of 4 pellets per trial. Rats were separately habituated to an unpaired audio cue (CS−; whichever cue was not used as CS+; 2-min duration). Rats were given 3 days of CS− only exposure (eight non-reinforced trials per session, 3 min variable ITI) following instrumental training (see below). Conditioning was measured by comparing the rate of food-cup approach between CS onset and the first pellet delivery (to exclude unconditioned feeding activity) to the rate of approach during the Pre-CS period.

#### Instrumental training

Rats then received 9 days of instrumental lever-press training. In each session, rats had continuous access to the lever, which could be pressed to deliver food pellets into the food cup. The schedule of reinforcement was adjusted over days from continuous reinforcement (CRF) to increasing random intervals (RI), such that reinforcement only became available once a randomly determined interval had elapsed since the last reinforcer delivery. Rats received 2 days of CRF, 1 day each of RI-15s and RI-30s, and 6 days of training with RI-60s. Each session was terminated after 30 min or after 20 rewards deliveries.

#### Pavlovian-instrumental transfer test

After the last instrumental training session, rats were given a session of Pavlovian (CS+) training, identical to initial training, and 3 sessions of CS− training. They were then given a 30 min extinction session, during which lever presses were recorded but had no consequence (i.e., no food or cues). On the next day, rats were given a PIT test, during which the lever was continuously available but produced no rewards. Following 8 min of extinction, the CS+ and CS− were each presented four times (2 min per trial) in pseudorandom order and separated by a fixed 3-min interval. Rats received CNO (5 mg/kg, i.p.) or vehicle (5% DMSO in saline) injections 30 min prior to testing. They underwent a second test following retraining, which consisted of two sessions of instrumental retraining (RI-60s), one session each of CS+ and CS− retraining, and one 30-min extinction session, as described above. The alternative drug pretreatment was administered prior to this second test (counterbalanced across groups).

### Histology

Rats were deeply anesthetized with pentobarbital sodium and transcardially perfused with PBS, followed by 4% PFA. Brains were removed and postfixed overnight in 4% PFA at 4°C, transferred to 30% sucrose, and then sectioned into 40-μm-thick coronary brain slices that were stored in cryoprotectant solution. The expression of DREADD-mCherry or GFP were immunohistochemically amplified using antibodies directed against mCherry or GFP. Tissue was first incubated in 3% normal donkey serum PBS plus Triton X-100 (PBST; 1 h) and then in primary antibodies in PBST at 4°C for 48 h using rabbit anti-DsRed (mCherry tag; 1:1,000; Clontech; 632496), or mouse anti-GFP (1:1,500, Life Technologies; A-11120) antibodies. Sections were incubated for 2 h at room temperature in fluorescent conjugated secondary antibodies Alexa Fluor 594 goat anti-rabbit (DsRed; 1:500; Invitrogen; A11037) or (Alexa Fluor 488 goat anti-mouse (GFP; 1:500; Invitrogen; A10667). Sections were mounted with mounting medium with DAPI (Vectashield) and were imaged with a 10 × objective on a fluorescence microscope (Leica) to validate viral expression. Two rats (one hM4Di and one hM3Dq) were omitted from analysis due to inadequate viral expression in the dmPFC.

### Data analysis

Statistical analysis was conducted in SPSS (v. 28.0.1), with alpha set at *p* < 0.05. Repeated measures ANOVAs were performed using relevant within-subjects factors such as Drug and Cue. For Pavlovian training and PIT testing, difference scores were computed by subtracting baseline response rates (responses per minute) during 2-min Pre-CS periods from response rates during CS periods. We focused on three distinct behavioral measures for PIT testing, the overall rate of lever pressing, the rate of initiating new bouts of lever pressing, and the rate of initiating new bouts of spontaneous (press-independent) food-port entry behavior. To identify new bouts of behavior, we assessed the distribution of inter-response times (IRTs) separating all responses performed during PIT testing (both full sessions), focusing on press-press, entry-entry, press-entry, and entry-press transitions with IRTs less than or equal to 10 s. Each distribution was normalized within-subject by dividing the number of IRTs in each 0.1-s bin by the total number of IRTs in the distribution.

## Results

**FIGURE 1:**
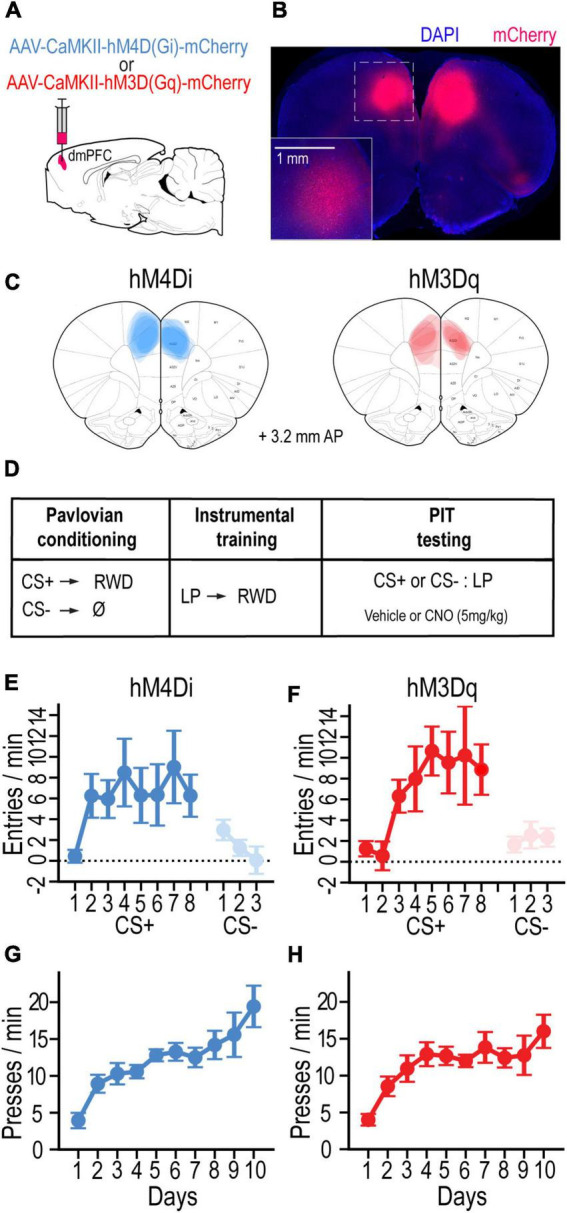
**(A)** Schematic of AAV strategy for expressing hM4Di or hM3Dq in dmPFC. **(B)** Coronal section showing representative mCherry-labeled DREADD expression. **(C)** Localization of DREADD expression for rats in hM4Di and hM3Dq groups (adapted from Paxinos and Watson, 2014). **(D)** Schematic of experimental design. **(E,F)** Mean cue-evoked food-port entry rate (entries/min; CS – Pre; ± SEM) during Pavlovian conditioning sessions with the CS+ and CS– for hM4Di **(E)** and hm3Dq **(F)** groups. **(G,H)** Mean rate of lever pressing (presses/min; ± SEM) during instrumental conditioning sessions for hM4Di **(G)** and hm3Dq **(H)** groups.

To investigate the contributions of the dmPFC to Pavlovian incentive motivation, AAV vectors were bilaterally injected into the dmPFC to locally express the inhibitory G-protein-coupled designer receptor hM4Di (*n* = 7) or the excitatory receptor hM3Dq (*n* = 7) in separate groups of rats (visualized with mCherry; Figures 1A–C). After recovering from surgery, rats were food deprived and trained on a standard PIT protocol (Figure 1D). Rats first received Pavlovian conditioning to associate an auditory cue (CS+) with a food-pellet reward. Both groups readily learned to enter the food-port during CS+ trials and withhold this response during CS− trials. By the final day of training with each cue, the CS+ increased food-port entries (CS – pre-CS) more than the CS− in groups hM4Di [*F*_(1, 6)_ = 6.00, *p* < 0.05] and hM3Dq [*F*_(1, 6)_ = 11.67, *p* < 0.014] (Figures 1E,F). Rats then received instrumental conditioning in the absence of the cue to learn that pressing a lever would earn the food-pellet reward. Both groups hM4Di [*F*_(9, 54)_ = 9.83, *p* < 0.001] and hM3Dq [*F*_(9, 54)_ = 8.09, *p* < 0.001] rapidly increased their rate of lever pressing over training days (Figures 1E,F).

**FIGURE 2:**
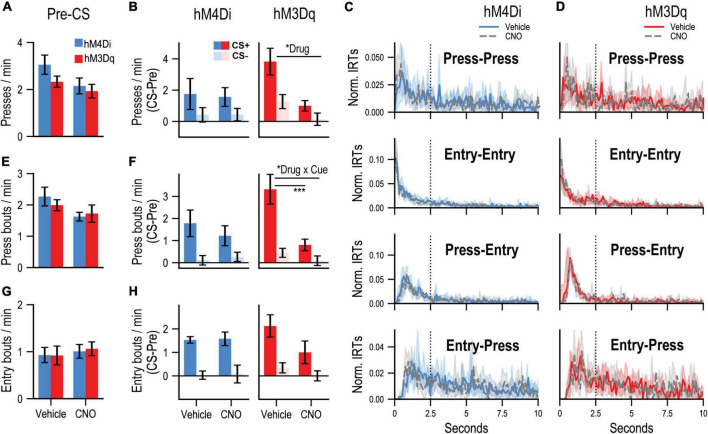
**(A)** Mean rates (responses/min; ± SEM) of lever pressing during Pre-CS (baseline) periods of the PIT test. **(B)** Mean rates (responses/min) of cue-evoked (CS – Pre; ± SEM) lever pressing at test. **(C,D)** Normalized inter-response time (IRT) distributions (<10 s; central tendency ± 95% confidence intervals; plotted over 0.1-s bins) for four possible response sequences: **Press-Press**, **Entry-Entry**, **Press-Entry**, and **Entry-Press**. Dotted vertical line indicates the 2.5-s cutoff used for bout analysis (refer to main text). Data are separately plotted for hM4Di **(C)** and hM3Dq **(D)** AAV group and drug condition as indicated. **(E)** Mean rates (responses/min; ± SEM) of press bouts during Pre-CS (baseline) periods of the PIT test. **(F)** Mean rates (responses/min) of cue-evoked (CS – Pre; ± SEM) press bouts at test. **(G)** Mean rates (responses/min; ± SEM) of spontaneous (press-independent) food-port entry bouts during Pre-CS (baseline) periods of the PIT test. **(H)** Mean rates (responses/min) of cue-evoked (CS – Pre; ± SEM) food-port entry bouts at test. “Drug” refers to significant main effects of Drug, and “Drug × Cue” refers to a significant Drug × Cue interaction. The bar below indicates a significant simple effect of Drug for the CS+ condition. *p < 0.05, ***p < 0.001.

PIT testing was then conducted to assess the motivational influence of the CS+ on instrumental performance. During each PIT test, the lever was available, but unrewarded, and each cue was presented in pseudorandom order. Rats received two tests, one following CNO and one following vehicle (counterbalanced for order) to determine the effects of dmPFC inhibition (CNO in hM4Di group) or stimulation (CNO in hM3Dq group) on PIT performance. While pre-CS press rates (Figure 2A) appeared to be slightly lower after CNO treatment, no reliable effect of drug was found for group hM4Di [*F*_(1, 6)_ = 3.28, *p* = 0.12] or group hM3Dq [*F*_(1, 6)_ = 0.48, *p* = 0.52], suggesting that these treatments did not induce gross alterations in baseline task performance. Difference scores (CS – pre-CS) were computed to isolate CS-related changes in responding (Figure 2B). While group hM4Di showed a tendency to increase their rate of lever pressing during CS+ relative to CS− trials, this effect did not reach significance [Cue: *F*_(1, 6)_ = 3.053, *p* = 0.098], making it difficult to determine whether their performance depended on associative learning or a non-associative processes such as pseudoconditioning. Regardless, these cue-related changes in press rate were not reliably altered by CNO administration in group hM4Di [Drug: *F*_(1, 6)_ = 0.003, *p* = 0.96; Drug × Cue interaction: *F*_(1, 6)_ = 0.003, *p* = 0.96]. In contrast, group hM3Dq showed a preferential elevation in lever pressing during CS+ relative to CS− [Cue: *F*_(1, 6)_ = 8.97, *p* = 0.008] and were also sensitive to CNO treatment, which generally attenuated cue-related lever pressing [Drug: *F*_(1, 6)_ = 12.01, *p* = 0.0028]. However, this drug effect did not significantly interact with cue type [Drug × Cue interaction: *F*_(1, 6)_ = 2.22, *p* = 0.15], which further complicates data interpretation since it remains unclear if stimulating the dmPFC specifically interrupted the acquired motivational influence of the CS+ or whether it had a more general effect. It is also worth noting that although group hM3Dq appeared to show a more pronounced increase in pressing during the CS+ than group hM4Di under control (vehicle) conditions, this difference was not significant [t(12) = 1.41, *p* = 0.18, two-tailed independent *t*-test] and likely reflects random between-subjects variability.

Given these issues, we analyzed the microstructure of lever pressing to more directly assay the response-*instigating* influence of the reward-paired cue, which is a hallmark of Pavlovian incentive motivation (Rescorla and Solomon, 1967; Bindra, 1978). Instrumental behavior is organized into continuous bouts of lever pressing that are separated by occasional pauses, and previous work indicates that the rate of bout initiation provides a more selective readout of motivation than the overall rate of lever pressing, which is influenced by other learning and performance factors (Shull et al., 2001, 2004; Shull and Grimes, 2003; Shull, 2004; Johnson et al., 2009; Brackney et al., 2011). By plotting the distribution of inter-response-times in a given test it is possible to identify new bouts of lever pressing, which are initiated after long IRTs, and can be readily distinguished from higher-frequency, within-bout lever presses, which are separated by short IRTs. Inspection of the IRT distribution for all lever presses performed by groups hM4Di (Figure 2C, Press-Press) and hM3Dq (Figure 2D, Press-Press) during PIT test sessions confirmed that this behavior was indeed organized into bouts of high-frequency pressing, reflected by the distinct cluster IRTs in the 0.5–2 s range. We therefore defined bout-initiating presses as those occurring at least 2.5 s after the last press, to avoid misclassifying within-bout presses. Previous studies have used similar cut-off values (Reed, 2011; Wassum et al., 2013; Smith et al., 2021) and indicate that such bout analyses are robust to variation in this parameter (Mellgren and Elsmore, 1991; Shull et al., 2002). Presses that occurred within 2.5 s of a food-port entry (Figures 2C,D and Entry-Press), regardless of the timing of the last lever press, were also categorized as bout-initiating presses as they represent a return to instrumental reward-seeking behavior.

During pre-CS periods, CNO administration did not significantly alter the rate at which new bouts of pressing were initiated (Figure 2E) in group hM4Di [Drug: *F*_(1, 6)_ = 3.66, *p* = 0.10] or group hM3Dq [Drug: *F*_(1, 6)_ =.37, *p* = 0.57]. Cue-elicited changes in bout initiation (CS – pre-CS, Figure 2F) were greater during CS+ trials in group hM4Di [Cue: *F*_(1, 6)_ = 6.29, *p* = 0.046] and group hM3Dq [Cue: *F*_(1, 6)_ = 12.32, *p* = 0.013], indicating that this measure was indeed more sensitive to the acquired motivational properties of the reward-predictive cue. CNO administration did not significantly alter cue-related bout initiation in group hM4Di [Drug: *F*_(1, 6)_ = 0.16, *p* = 0.71; Cue × Drug: *F*_(1, 6)_ = 0.41, *p* = 0.55]. In contrast, this behavior was strongly suppressed by CNO in group hM3Dq [Drug: *F*_(1, 6)_ = 17.63, *p* = 0.006; Cue × Drug interaction: *F*_(1, 6)_ = 13.77, *p* = 0.01], an effect that was limited to CS+ [*F*_(1, 6)_ = 18.67, *p* = 0.005] but not CS− [*F*_(1, 6)_ = 1.98, *p* = 0.21] trials.

We also examined how these treatments affected Pavlovian cue-elicited food-port entry behavior. Food-port entries were often performed in concert with ongoing instrumental behavior, as coordinated press-entry sequences (Marshall and Ostlund, 2018; Halbout et al., 2019; Marshall et al., 2020), which is reflected by the preponderance of short IRT Press-Entry sequences at test (Figures 2C,D and Press-Entry). The distribution of Entry-Entry IRTs (Figures 2C,D and Entry-Entry) indicated that these responses, like Press-Press sequences, were also clustered into discrete bouts of high frequency behavior (short IRTs). We therefore defined new bouts of spontaneous (press-independent) food-port entry behavior as entries that occurred at least 2.5 s after either the last entry or lever-press response.

We found that, during the pre-CS period, CNO administration did not alter the initiation of new food-port entry bouts in group hM4Di [Drug: *F*_(1, 6)_ = 0.028, *p* = 0.87] or group hM3Dq [Drug: *F*_(1, 6)_ =.76, *p* = 0.42; Figure 2G]. The CS+ was also more effective that the CS− at eliciting new entry bouts (Figure 2H) in groups hM4Di [Cue: *F*_(1, 6)_ = 30.97, *p* = 0.001] and hM3Dq [Cue: *F*_(1, 6)_ = 24.27, *p* = 0.003]. While cue-evoked food-port entry bouts appeared to be slightly attenuated by CNO in the hM3Dq group, this effect was not significant [Drug: *F*_(1, 6)_ = 2.59, *p* = 0.16; Drug × Cue: *F*_(1, 6)_ = 1.31, *p* = 0.30], nor was there a significant influence of CNO in group hM4Di [Drug: *F*_(1, 6)_ = 0.17, *p* = 0.70; Drug × Cue: *F*_(1, 6)_ = 0.002, *p* = 0.96].

**FIGURE 3:**
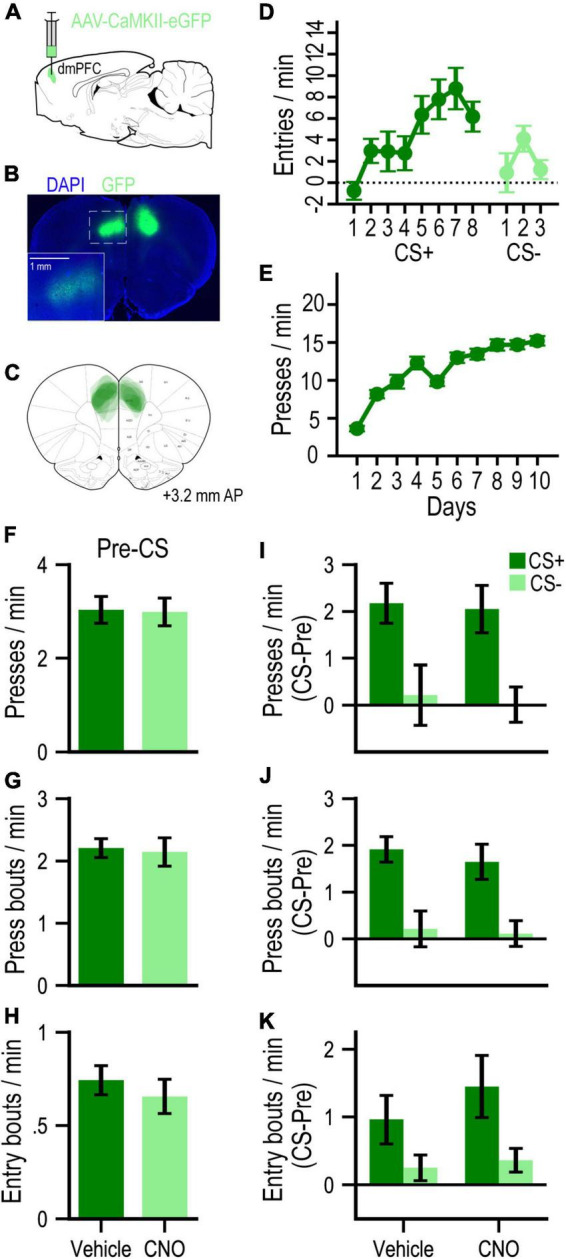
**(A)** Schematic of AAV strategy for expressing GFP in dmPFC. **(B)** Representative GFP expression. **(C)** Localization of expression for all rats in this experiment. **(D)** Mean cue-evoked food-port entry rate (entries/min; CS – Pre; ± SEM) during Pavlovian conditioning sessions with the CS+ and CS– for GFP group. **(E)** Mean rate of lever pressing (presses/min; ± SEM) during instrumental conditioning sessions for GFP group. **(F–H)** Mean rates (responses/min; ± SEM) of lever pressing **(F)**, press bouts **(G)**, and spontaneous (press-independent) food-port entry bouts **(H)** during Pre-CS (baseline) periods of the PIT test. **(I–K)** Mean rates (responses/min) of cue-evoked (CS – Pre; ± SEM) lever pressing **(I)**, press bouts **(J)**, and food-port entry bouts **(K)** at test.

To assess potential non-specific (DREADD-independent) behavioral effects of CNO administration, we conducted a separate experiment with GFP-only control rats (*n* = 10; Figures 3A–C). These rats readily learned to approach the food-port during Pavlovian conditioning (Figure 3D), such that by the last day of training with each cue, the CS+ elicited higher rates of entry than the CS− [*F*_(1, 9)_ = 17.31, *p* = 0.002]. They subsequently learned to press the lever for food pellets over instrumental training days [*F*_(9, 81)_ = 36.75, *p* < 0.001; Figure 3E]. During PIT testing, we found no effect of CNO on baseline (pre-CS) rates of pressing (*F* < 1, *p* = 0.94; Figure 3F), bouts of pressing (*F* < 1, *p* = 0.86; Figure 3G) or bouts of food-port entry (*F* < 1, *p* = 0.57; Figure 3H). These measures were all selectively elevated on CS+ vs. CS− trials [press rate: *F*_(1, 9)_ = 21.83, *p* = 0.001; press bout rate: *F*_(1, 9)_ = 24.36, *p* < 0.001; entry bout rate: *F*_(1, 9)_ = 7.20, *p* = 0.025; Figures 3I–K, respectively], in a manner that was not significantly affected by CNO administration (all Drug effects and Drug × Cue interactions, *F*’s ≤ 1.25, *p*’s ≥ 0.30).

## Discussion

The current study examined the role of the dmPFC in Pavlovian incentive motivation. We found that stimulating the dmPFC (via CNO administration in the hM3Dq group) during PIT testing led to a pronounced disruption of cue-motivated lever pressing, whereas inhibiting the dmPFC (via CNO administration in the hM4Di group) had no reliable behavioral effects. These findings suggest that the dmPFC is capable of regulating Pavlovian incentive motivation but is not required for its expression. Moreover, no behavioral effects of CNO administration were observed in a reporter-only control group, confirming that this treatment did not have non-specific, DREADD-independent effects on PIT performance. Interestingly, dmPFC stimulation did not significantly alter Pavlovian cue-evoked food-port approach behavior, suggesting this structure is preferentially involved in regulating the motivational influence of reward-associated cues on instrumental reward seeking, rather than by exerting widespread control over all motor behavior.

The lack of effect of dmPFC inhibition on PIT expression would seem to be at odds with theories assigning this structure a motivational function (Paus, 2001; Stuss and Alexander, 2007; Holroyd and Yeung, 2012). Indeed, the dmPFC is closely connected with multiple brain regions implicated in PIT, including the ventral tegmental area, nucleus accumbens, dorsal striatum, mediodorsal thalamus, and basolateral amygdala (Cartoni et al., 2016; Corbit and Balleine, 2016). Moreover, a large proportion of dmPFC neurons responds to reward-predictive cues (Takenouchi et al., 1999; Otis et al., 2017), and encodes motivationally relevant parameters such as the magnitude, probability, and proximity of reward (Shidara and Richmond, 2002; Amiez et al., 2006; Kennerley et al., 2011; Toda et al., 2012). The dmPFC has also been implicated in other behavioral tests thought to engage Pavlovian incentive motivation, such as cue-induced reinstatement of drug-seeking behavior (Moorman et al., 2015; Feltenstein et al., 2021) and discriminative stimulus-elicited food-seeking behavior (Ishikawa et al., 2008).

However, our finding that the dmPFC is not critical for the expression of cue-elicited incentive motivation is consistent with previous PIT studies. For instance, Cardinal et al. (2003) found that rats with permanent excitotoxic lesions of the anterior cingulate were unimpaired on a simple (single-reward) PIT task similar to the one used in the current study. There is evidence that this version of the PIT task is predominantly driven by a non-specific or general appetitive arousal process that is capable of enhancing reward-seeking behavior broadly, regardless of which reward is predicted, though more direct measures of this so-called general PIT effect have been developed (Corbit and Balleine, 2016). Around the same time, Corbit and Balleine (2003) found that excitotoxic lesions of the nearby prelimbic cortex left intact the outcome-specific PIT effect, which measures a distinct influence of reward-predictive cues, namely their ability to bias action selection to promote the pursuit of a particular outcome (Corbit and Balleine, 2016). Interpreting such findings is complicated since permanent brain lesions may allow for functional compensation by neural circuitry that was intact during initial training sessions. The chemogenetic inhibition strategy used here, which was previously shown to reduce neuronal dmPFC neuronal activation and associated behaviors (Giannotti et al., 2018; Schmidt et al., 2019; Stolyarova et al., 2019), avoids this issue and bolsters the conclusion that the dmPFC is not a critical mediator of PIT expression.

While dmPFC inhibition did not impact PIT performance, stimulating the dmPFC attenuated this effect, which we suggest reflects this structure’s capacity to exert inhibitory control over cue-motivated behavior. This is in line with previous findings that disrupting dmPFC function can weaken inhibitory control (Bussey et al., 1996; Muir et al., 1996; Broersen and Uylings, 1999; Narayanan and Laubach, 2006; Kamigaki and Dan, 2017; Hvoslef-Eide et al., 2018; Brockett et al., 2020; Li et al., 2020; Terra et al., 2020). However, these previous findings on their own do not address whether the dmPFC is specifically involved in regulating the expression of Pavlovian incentive motivation. This form of motivation, which is thought to drive impulsive and compulsive behaviors (Robinson and Berridge, 2008; Bari and Robbins, 2013), is not selectively probed in conventional tests of inhibitory control, which focus on measures such as premature, uncued responding (e.g., 5-choice serial reaction time task) or responding to inappropriate, non-reinforced cues (e.g., go/no-go or stop-signal tasks). While such responses may be motivated by prevailing reward-predictive cues, it is equally plausible that they are simply learned motor responses (e.g., conditioned reflexes or habits). This distinction is important as it is believed that behavioral/motor and emotional/motivational processes are regulated by separate neural systems (Bari and Robbins, 2013; Freeman et al., 2014).

Previous studies have shown that activating hM3Dq receptors on dmPFC neurons increases their spontaneous and evoked activity (Hart et al., 2020). If neural activity in the dmPFC mediates a top-down inhibitory control function over Pavlovian incentive motivation, as hypothesized, then activating dmPFC neurons via hM3Dq-stimulation should suppress cue-motivated behavior, as reported here. However, it is also possible that stimulating dmPFC activity interfered with ongoing incentive motivational processing in downstream sites in a manner that may not reflect a normal function of that circuit. Further research will be needed to assess this possibility and determine if the dmPFC is in fact normally recruited to adaptively suppress maladaptive cue-motivated behavior. Importantly, while dmPFC inhibition did not alter PIT expression in the current study, the task used here was designed to assay an adaptive form of cue-motivated behavior and is therefore unlikely to engage of top-down control circuitry (Ostlund and Marshall, 2021).

In this context, it is useful to compare the current findings with a recent study examining the role of the nearby prelimbic cortex on regulating the expression of Pavlovian conditioned approach behavior (Campus et al., 2019), which focused on rats’ tendency to sign-track (approach the reward-predictive cue) vs. goal-track (approach the food-port). A compelling case has been made that sign-tracking behavior represents a motivational response to the reward-predictive cue, whereas goal-tracking is the product of a cognitive, cue-evoked reward expectancy (Robinson et al., 2018). Using a bidirectional chemogenetic strategy similar to the one used here, Campus et al. (2019) found that inhibiting an anatomically defined subset of prelimbic neurons projecting to the paraventricular thalamus caused goal-trackers to sign-track (presumably by disinhibiting the Pavlovian incentive motivational system), and that stimulating these neurons caused sign-trackers to goal-track (presumably by inhibiting the motivational system). The findings are generally compatible with those reported here, though we targeted a more dorsal and less anatomically restricted population of medial prefrontal neurons for manipulation. Moreover, whereas Campus et al. (2019) conducted chemogenetic manipulations throughout training and test sessions, precluding conclusions about whether learning or performance processes were altered, our manipulations were restricted to test sessions to focus exclusively on performance processes.

The current study used response microstructure to isolate the tendency for reward-paired cues to instigate new bouts of lever pressing. There is growing evidence that reinforcement and motivational processes selectively influence the rate at which animals initiate new bouts of reward seeking (Shull et al., 2001, 2004; Shull and Grimes, 2003; Shull, 2004; Johnson et al., 2009; Brackney et al., 2011) and consumption (Marshall et al., 2018; D’Aquila et al., 2019). In the current study, the press bout rate measure proved to be useful for revealing associatively-mediated (i.e., CS+ specific) changes in lever press performance during PIT, which was critical for showing that the suppressive effect of dmPFC stimulation on cue-related lever pressing was specific to the CS+. This utility of bout analyses is also apparent in previous studies examining the neural mechanisms of PIT (Marshall and Ostlund, 2018; Halbout et al., 2019; Marshall et al., 2020). For instance, during cue presentations, bout-initiating lever presses are preceded phasic dopamine release in the nucleus accumbens (Wassum et al., 2013) and phasic glutamate release in the basolateral amygdala (Malvaez et al., 2015). Importantly, these neurochemical responses do not typically precede the execution of other (within-bout) lever presses and have an increased likelihood of occurring during motivationally-relevant, reward predictive cues.

The current findings reveal the dmPFC’s capacity to regulate expression of Pavlovian incentive motivation. Since the current study used only male rats as subjects, further work will be needed to explore potential sex differences. Future studies will also be needed to determine whether and under which conditions the dmPFC is enlisted to flexibly suppress cue-motivated reward seeking, such as when this behavior might interfere with more adaptive reward retrieval activity (Marshall et al., 2020; Marshall and Ostlund, 2021; Ostlund and Marshall, 2021). It will also be important to identify the downstream circuitry through which the dmPFC exerts its suppressive influence over cue-motivated behavior, particularly as dysfunction in this circuitry may contribute to pathological forms of motivated behavior in addiction and other psychiatric disorders (Goldstein and Volkow, 2011; Bari and Robbins, 2013). The dmPFC may exert this influence by dampening incentive processes at projection sites or by recruiting additional components implicated in regulating motivated behavior including the paraventricular thalamus (Campus et al., 2019), subthalamic nucleus (Li et al., 2020), or striatal cholinergic interneuron system (Collins et al., 2016, 2019).

## Data availability statement

The raw data supporting the conclusions of this article will be made available by the authors, without undue reservation.

## Ethics statement

All experimental procedures were approved by the UC Irvine Institutional Animal Care and Use Committee (IACUC) and conducted in accordance with the National Research Council Guide for the Care and Use of Laboratory Animals.

## Author contributions

BH, SO, and KW contributed to the conception and design of the study and were responsible for writing the manuscript. BH and CH performed the experiments. BH and SO analyzed the data. All authors approved the submitted version of the manuscript.
